# Supplementary figures and images for: Zinc protection of fertilized eggs is an ancient feature of sexual reproduction in animals
Source: PLoS Biol. 2020 Jul 31;18(7):e3000811. doi: 10.1371/journal.pbio.3000811 (PMC7423145; doi:10.1371/journal.pbio.3000811)

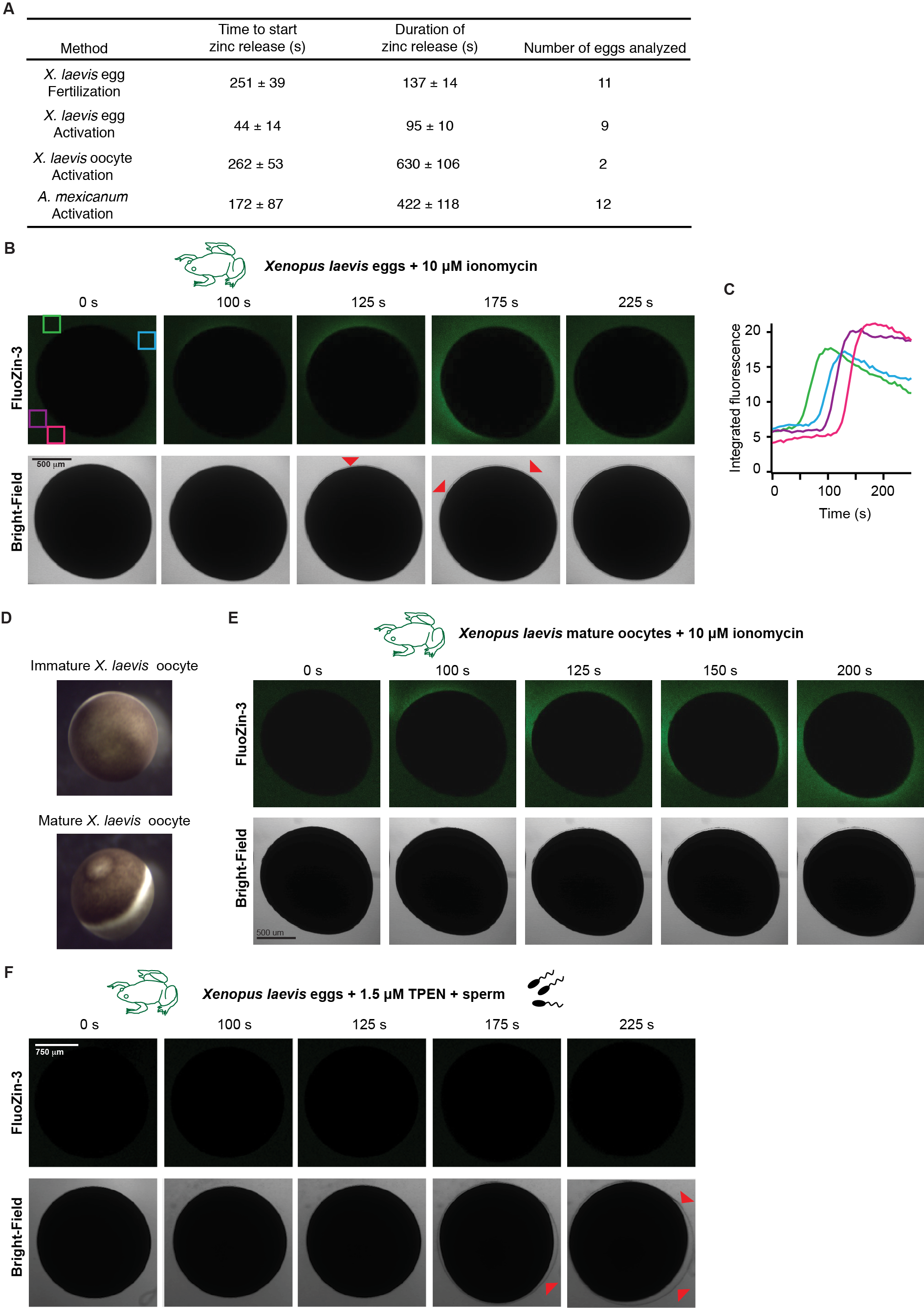

Supplement: S1 Fig — (A) Kinetics of zinc release in activation of X. laevis and A. mexicanum eggs and oocytes. (B) Parthenogenic activation of X. laevis eggs (N = 9 eggs, 5 trials) or in vitro matured oocytes (E; N = 9 eggs, 3 trials) with 10 μM ionomycin in the presence of FluoZin-3 also induced zinc exocytosis. Red arrowheads highlight the lifting of the fertilization envelope (B, F). (C) Changes in FluoZin-3 fluorescence upon parthenogenic activation were detected by region of interest analysis. Integrated fluorescence relative to time of ionomycin addition, detected by region of interest analysis (indicated by colored boxes in upper left image). (D) Representative images of immature and in vitro matured oocytes. (F) Treatment of X. laevis eggs in FlouZin-3 with the zinc chelator TPEN abolished fertilization-induced zinc release (N = 8 eggs, 2 trials). For full source data, see S1 Data. (PNG) [file pbio.3000811.s001.png]

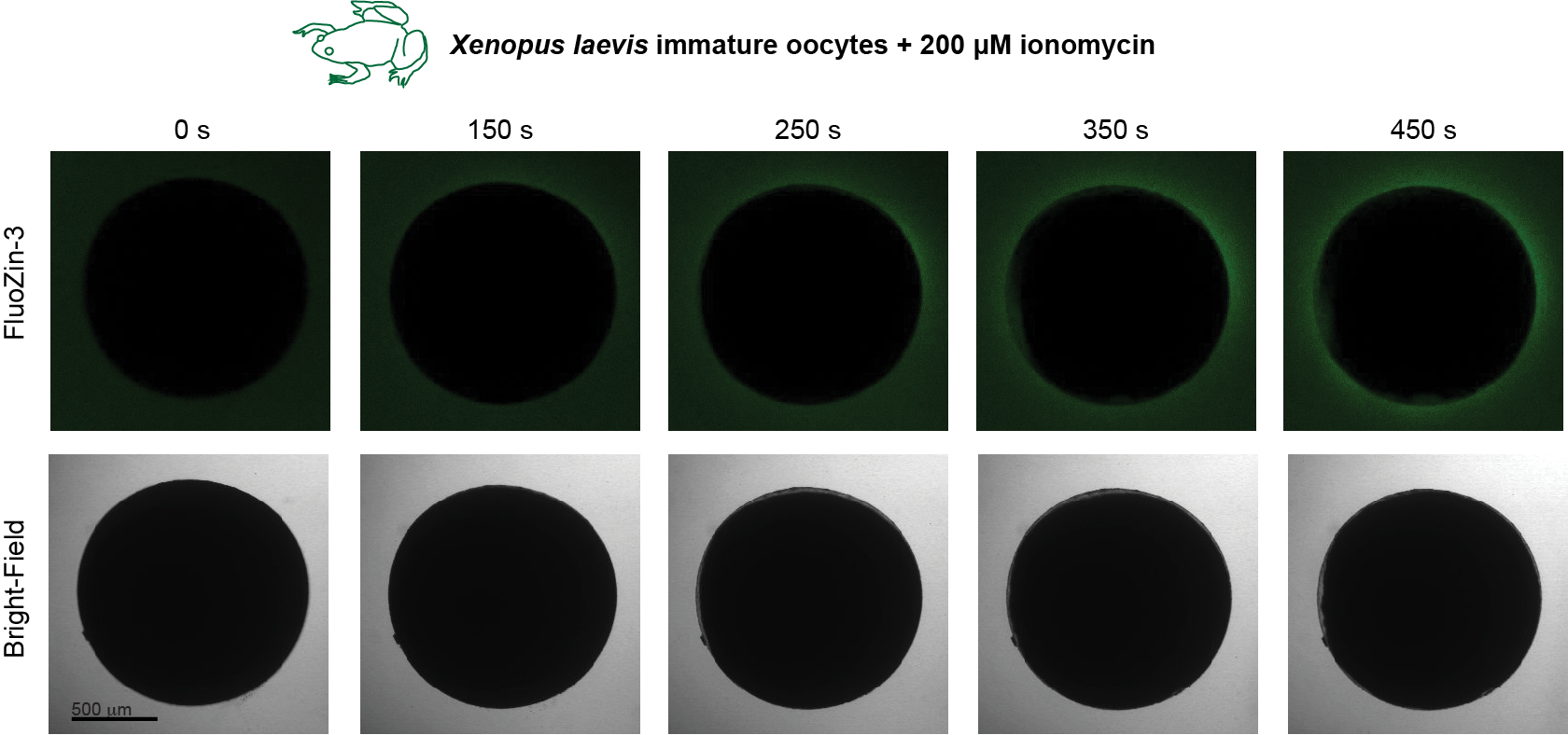

Supplement: S2 Fig — Zinc released upon activation of immature X. laevis oocytes with 200 μM ionomycin (N = 15 eggs, 7 trials). (PNG) [file pbio.3000811.s002.png]
